# Pre-treatment ^18^F-choline PET/CT is prognostic for biochemical recurrence, development of bone metastasis, and cancer specific mortality following radical local therapy of high-risk prostate cancer

**DOI:** 10.1186/s41824-018-0034-2

**Published:** 2018-08-07

**Authors:** Henrik Kjölhede, Helén Almquist, Kerstin Lyttkens, Ola Bratt

**Affiliations:** 1 Department of Urology, Institute of Clinical Sciences, Sahlgrenska Academy at the University of Gothenburg, Sahlgrenska University Hospital, Göteborg, Sweden; 2 Center for Medical Imaging and Physiology, Skåne University Hospital, Lund, Sweden

**Keywords:** PET/CT, Choline, Prostate cancer, Prognosis

## Abstract

**Background:**

The aim of this study was to determine whether lymph node metastasis on pre-treatment ^18^F-choline PET/CT is an independent prognostic factor for biochemical recurrence (BCR), skeletal metastasis, and cancer specific mortality (CSM), after radical local treatment (radical prostatectomy and/or radiotherapy) in men with high-risk prostate cancer. Medical records were reviewed for men with newly diagnosed high-risk prostate cancer who had pre-treatment ^18^F-choline positron emission tomography fused with computed tomography (PET/CT) scan for primary metastasis staging.

**Results:**

Of 174 eligible men, 124 met the criteria for inclusion. The PET/CT scan was negative for metastasis in 97 (78%) men, inconclusive in 15 (12%), and positive in 12 (10%). The men with a positive PET/CT scan had significantly shorter time to BCR (*p* = 0.02), time to skeletal metastasis (*p* = 0.002), and time to prostate cancer specific death (*p* < 0.001). On multivariable Cox regression analysis, including also tumour stage, Gleason score, and PSA, a non-negative PET/CT scan was the only significant covariate for time to BCR (HR 2.6, 95% CI 1.3–5.5) and time to skeletal metastasis (HR 2.7, 95% CI 1.3–5.9).

**Conclusions:**

In men with a newly diagnosed high-risk prostate cancer and a negative or inconclusive bone scan, ^18^F-choline uptake on PET/CT suggestive metastasis was associated with recurrence, progression to distant metastasis, and prostate cancer death. This strongly indicates that the choline uptakes represented metastasis and not false positive findings.

## Background

Men with recently diagnosed prostate cancer face very different outcomes, and the presence or absence of metastases is one of the most important prognostic factors (Moschini et al. 2016). Positron emission tomography fused with computed tomography (PET/CT) with ^18^F-choline as tracer has been evaluated in several studies and shown to accurately detect prostate cancer metastasis (Evangelista et al. 2013). We have previously reported on ^18^F-choline PET/CT for primary staging of high-risk prostate cancer (Kjölhede et al. 2012, 2014, 2017): ^18^F-choline PET/CT indicated metastasis in 20–39% of men with high-risk prostate cancer, and the specificity of findings of regional lymph node metastasis was high at 92% (95% CI 0.82–0.97). A limitation of all these studies is that the metastases suggested by choline uptake outside the pelvic lymphadenectomy template were not verified with histology. An alternative to histopathological verification is to determine whether the men with suspicious metastasis on ^18^F-choline PET/CT have a worse prognosis in terms of biochemical recurrence (BCR), skeletal metastasis, and prostate cancer-specific mortality (CSM). This has been done with positive results in the setting of BCR after radical prostatectomy (Giovacchini et al. 2013, 2015; Colombié et al. 2015; Zattoni et al. 2017), but not for primary staging at the time of diagnosis.

We hypothesized that the men with choline uptake on PET/CT suggestive of metastasis in our previous study would have worse prognosis than those who had a negative scan, which would strongly indicate that the uptakes represented metastasis and not false positive findings. The aim of this study was thus to re-evaluate the subjects in our previous studies to determine whether pre-treatment ^18^F-choline PET/CT findings are independent prognostic factors for BCR, skeletal metastasis, and CSM, after radical local treatment (radical prostatectomy and/or radiotherapy) in men with high-risk prostate cancer.

## Methods

### Patients and ethics

This study included all men under the age of 75 years that had a ^18^F-choline PET/CT at Skåne University Hospital, between 27 February 2008 and 8 November 2011, who were considered for radical local treatment for a newly diagnosed, biopsy verified, high-risk prostate cancer, and had a normal or inconclusive ^99m^Tc-MDP planar bone scan. High-risk prostate cancer was defined as Gleason score 8–10 and/or prostate specific antigen (PSA) ≥ 20 ng/ml. Men who were treated with hormonal therapy before the PET/CT scan or who had a PSA ≥ 100 ng/ml were excluded. The clinical management of men after the PET/CT scan was decided by the referring urologist, often after discussing the findings with a member of the study group. For this sub-study, only the men who received curative therapy were analysed. During follow-up, imaging to assess skeletal metastasis, was done at the discretion of the referring urologist, usually due to rising PSA or newly developed symptoms. The study was approved by the Regional Ethical Review Board in Lund (LU552/2007).

### PET/CT imaging

The imaging protocol has been previously described in detail (Kjölhede et al. 2012). In summary, the PET/CT scans were acquired with an integrated PET/CT system (Philips Gemini TF, Philips Medical Systems, Cleveland, OH, USA) at the Centre for Medical Imaging and Physiology, Skåne University Hospital in either Lund or Malmö. Whole-body PET was acquired 1–1.5 h after intravenous injection of 4 MBq/kg ^18^F-fluorocholine with 2 min per bed position. A diagnostic quality CT scan was acquired immediately before the PET scan, with 1000 ml oral contrast given 60 min before the scan, and intravenous contrast given by an automatic injection pump. The CT scans were acquired in three phases: without intravenous contrast, in an arterial contrast phase, and in a portal contrast phase. All PET/CT scans were interpreted by both a nuclear medicine physician and a radiologist. Scans showing enlarged lymph nodes (> 1 cm short axis), or visually distinct ^18^F-choline uptake in more than one lymph node or in bone sites not corresponding to other pathology, were reported as positive. Scans showing a single, non-enlarged lymph node with ^18^F-choline uptake, or multiple lymph nodes with non-distinct uptake, were reported as inconclusive.

### Initial data acquisition

The men were prospectively enrolled in the study at the time of PET/CT. Clinical stage, Gleason score, and PSA level at the time of referral for PET/CT were collected retrospectively in 2012.

### Follow-up data acquisition

Date of BCR, date of decision to give salvage radiation therapy after radical prostatectomy, date of first confirmed bone metastasis, date of death, and cause of death were collected retrospectively from medical records. Date of BCR was defined as the date of analysis of confirmatory PSA ≥ 0.2 ng/ml after prostatectomy or nadir + 2 ng/ml after external beam radiation therapy, or as the date of decision to give salvage radiation therapy after radical prostatectomy if this was earlier. Date of first confirmed bone metastasis was defined as the date of performing imaging showing unequivocal bone metastasis. Time to events (BCR, skeletal metastasis, or death) was calculated from the date of prostatectomy or from the start of neoadjuvant hormonal therapy. Cause of death (prostate cancer or other cause) was determined by an independent monitor (JÖ and AL) blinded to the PET/CT results.

### Statistics

Kaplan-Meier survival analysis was performed for BCR, bone metastasis, and CSM, with log-rank test for differences in the outcome of men with a positive, negative, or inconclusive ^18^F-choline PET/CT scan. In a multivariable Cox regression analysis, biopsy Gleason score was dichotomized as ≤4 + 3 or ≥ 4 + 4 (ISUP grade ≤ 3 or ≥ 4), clinical stage as < or ≥ T3), PET/CT scans as negative or non-negative (i.e. positive or inconclusive), whereas serum PSA was analysed as a continuous variable. Men with suspected bone metastasis on the PET/CT scan were excluded from analysis of time to bone metastasis.

## Results

**Fig. 1:**
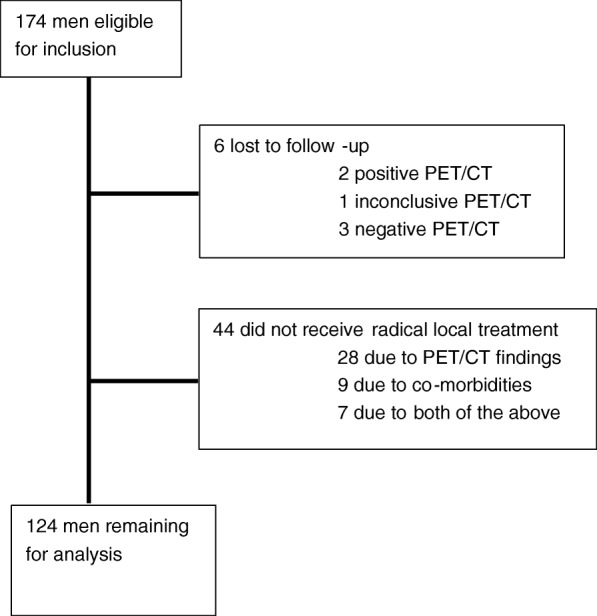
Inclusion and exclusion flow chart. Abbreviations: PET/CT, positron emission tomography fused with computed tomography

A total of 174 men with newly diagnosed high-risk prostate cancer had a ^18^F-choline PET/CT scan. Of these, 6 were lost to follow-up and 44 did not receive curative treatment (Fig. 1). The remaining 124 men had radical local treatment and were included in the study. Their clinical characteristics are presented in Table 1. The median time of follow-up was 75 (interquartile range 65–83) months. Thirty men (24%) experienced BCR and 6 (5%) were diagnosed with skeletal metastasis. Fifteen men (12%) died during follow-up: three (2%) from prostate cancer and 12 (10%) from other causes.

**Table 1:** The clinical characteristics of the 124 men who received radical local therapy

Age, yrs	
Mean (SD)	66.4 (6.1)
PSA, ng/ml	
Median (IQR)	22 (11–36)
Biopsy Gleason Score, n (%)	
3 + 3	9 (7)
3 + 4	25 (20)
4 + 3	16 (13)
4 + 4/3 + 5/5 + 3	30 (24)
4 + 5/5 + 4/5 + 5	44 (36)
Clinical tumor stage, n (%)	
Tx	1 (1)
T1	28 (23)
T2	44 (36)
T3	51 (41)
^18^F-choline PET/CT, n (%)	
Negative	97 (78)
Inconclusive	15 (12)
Positive	12 (10)
Local therapy	
Radical prostatectomy	28 (23)
Radiation therapy	96 (77)

*Abbreviations*: *SD* standard deviation, *PSA* prostate specific antigen, *IQR* inter-quartile range, *PET/CT* positron emission tomography fused with computed tomography

**Fig. 2:**
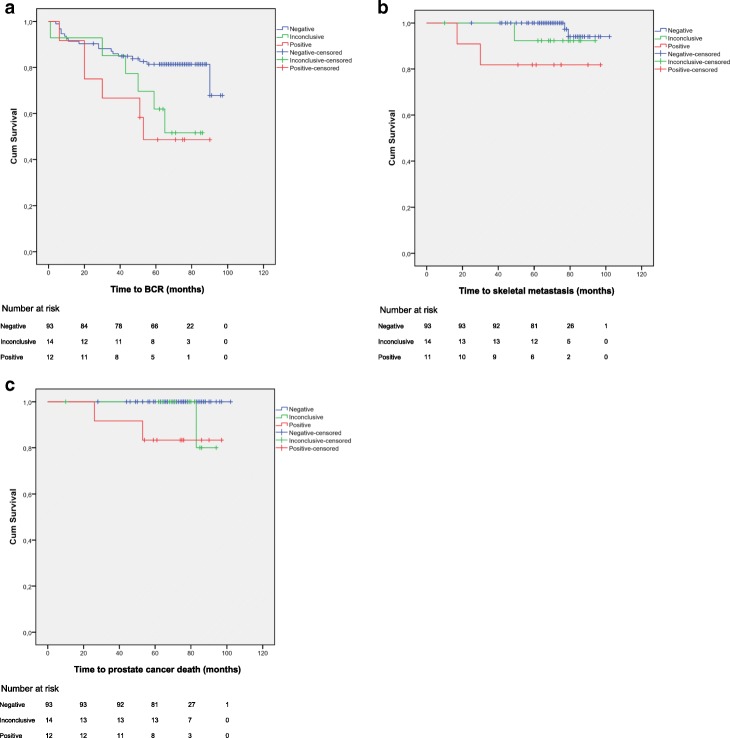
Kaplan-Meier curves of survival stratified according to ^18^F-choline PET/CT findings. Log-rank *p*-values are shown for differences between men with negative and positive findings. **a** Time to biochemical recurrence, *p* = 0.02. **b** Time to skeletal metastasis, *p* = 0.002. One man whose bone metastasis was confirmed by other imaging has been excluded from analysis. **c** Time to prostate cancer-specific death, *p* < 0.001. Abbreviations: PET/CT, positron emission tomography fused with computed tomography; BCR, biochemical recurrence

The PET/CT scan was negative in 97 (78%), positive in 12 (10%), and inconclusive in 15 (12%) of the 124 men. Of the 97 men who had a negative PET/CT scan, 19 (20%) were treated with radical prostatectomy and 78 (80%) with radiation therapy; 18 (19%) of these men had BCR, 2 (2%) were later diagnosed with skeletal metastasis. None of them died from prostate cancer, but 9 (9%) died of other causes. Of the 12 men with a positive PET/CT scan, 5 (42%) were treated with radical prostatectomy and 7 (58%) with radiation therapy; 6 (50%) of these men had BCR, 3 (25%) developed skeletal metastasis. Two of them (17%) died of prostate cancer, and 2 (17%) died of other causes. The positive findings were located in iliac lymph nodes only in seven men, in pelvic (including iliac) lymph nodes only in three men, and in bone in two men (one bone site in one man and two sites in the other). The man with one bone lesion also had a positive pelvic lymph node. BCR was observed in only one of five men with only two positive iliacal lymph nodes, but in five of seven men with more extensive metastasis. Six of the 15 (40%) men with inconclusive scans had BCR, 1 (7%) had skeletal metastasis, 1 (7%) died of prostate cancer, and 1 (7%) died of other causes. The men with a positive PET/CT scan had significantly shorter time to BCR (Fig. 2a), to skeletal metastasis (Fig. 2b), and to prostate cancer-specific death (Fig. 2c), compared to the men with a negative PET/CT scan (all log rank *p* < 0.02). Five-year BCR-free survival was 81.4% for men with a negative scan, 61.9% for men with an inconclusive scan, and 48.6% for men with a positive scan. Five-year skeletal metastasis-free survival was 100, 92.3, and 81.8%, respectively. Five-year prostate cancer-specific survival was 100, 100, and 83.3%, respectively.

On multivariable Cox regression analysis, a non-negative finding on ^18^F-choline PET/CT was the only significant covariate for both time to BCR and time to skeletal metastasis, while PSA, biopsy Gleason score, and clinical local tumour stage were all non-significant (Table 2). Time to prostate cancer specific death was not analysed due to the low number of events.

**Table 2:** Results of multivariable Cox regression analysis with hazard ratios for each variable for time to BCR and time to skeletal metastasis, with PSA as a continuous variable and Gleason score, local tumour stage and PET/CT findings dichotomized

Variable	Time to BCR		Time to skeletal metastasis	
	HR (95% CI)	p	HR (95% CI)	p
PSA	1.00 (0.98–1.02)	.96	1.00 (0.98–1.03)	.84
Gleason score ≥ 4 + 4	1.44 (0.57–3.66)	.44	1.16 (0.42–3.21)	.77
Local tumour stage ≥ cT3	0.86 (0.39–1.93)	.72	0.89 (0.38–2.10)	.79
Non-negative PET/CT	2.63 (1.26–5.49)	.01	2.73 (1.26–5.90)	.01

*Abbreviations*: *PSA* prostate specific antigen, *PET/CT* positron emission tomography fused with computed tomography, *BCR* biochemical recurrence, *HR* hazard ratio

## Discussion

The present study showed that ^18^F-choline PET/CT findings were significantly associated with recurrence, progression to distant metastasis, and prostate cancer death in men with newly diagnosed high-risk prostate cancer and a negative or inconclusive bone scan, which strongly indicates that the choline uptakes represented metastasis and not false positive findings. Men with a PET/CT scan suggesting metastasis had significantly shorter time to BCR, skeletal metastasis, and prostate cancer death than men with a negative scan. PET/CT findings were the only significant predictor of BCR and of skeletal metastasis on multivariable analysis.

Our results imply that the positive predictive value of choline uptake on PET/CT findings is high, not only in regional lymph nodes as previously reported, but also in non-regional lymph nodes and distant metastasis. This could help make informed decisions for men with positive choline PET/CT findings, especially in cases with more than one uptake site.

These results agree well with those of studies on choline PET/CT in the setting of BCR after radical prostatectomy. In an early study, Breeuwsma et al. found that men with a negative ^11^C-choline PET/CT scan at BCR after radical prostatectomy had both lower treatment rates and higher disease-specific survival than men with a positive scan (Breeuwsma et al. 2012). Similarly, Giovacchini et al. reported on a study of 195 men with rising PSA during androgen deprivation therapy for BCR after radical prostatectomy, in which men with a positive ^11^C-choline PET/CT scan were more than twice as likely to die of prostate cancer than men with a negative scan, even after adjusting for other prognostic factors in a multivariable analysis (Giovacchini et al. 2014). The same study group also reported on a study of men with BCR after radical prostatectomy who were not on hormonal treatment, in which the hazard ratio was 6.34 (95% CI 2.1–18.9) for CSM in men with positive ^11^C-choline PET/CT findings (Giovacchini et al. 2015). Colombié et al. developed a scoring system for ^18^F-choline PET/CT findings in men with BCR after any primary treatment, based on age and uptake values (Colombié et al. 2015). They found a median progression free survival of only 11 months in the men with the highest score, compared with 49 months in those with the lowest score – whose progression free survival was not significantly different from those with a negative PET/CT. Most recently, Zattoni et al. compared ^18^F-choline PET/CT, ^99m^Tc-MDP bone scan, and CT for detecting bone metastasis, and related the findings to time to progression, skeletal events, and overall survival (Zattoni et al. 2017). They found that ^18^F-choline PET/CT was the best predictor of all three end points. In conclusion, the results from the above-mentioned studies suggest that choline PET/CT provides more accurate prognostic information than other clinical variables for men with recurrence after previous local therapy. According to the results of the present study, this might also be the case for pre-treatment choline PET/CT findings.

The present results should not lead to the conclusion that all men with positive choline PET/CT findings should be deprived of local therapy to the prostate. While the 12 men in our study with positive PET/CT findings who had a radical prostatectomy or radiotherapy were likely highly selected, the median time to BCR was as long as 53 months, which suggests that the local therapy may have delayed disease progression.

There has in recent years been an increasing awareness of the potential benefit of treating the primary tumour in men with metastatic prostate cancer, particularly in those with oligometastatic disease (Rusthoven et al. 2016; Bayne et al. 2016; Steuber et al. 2017; Seisen et al. 2017). However, the studies reported so far have been either retrospective, with all the inherent biases that entails, or prospective case series without control subjects. Metastasis directed therapy in oligometastatic disease has also recently attracted attention (Osmonov et al. 2016; Pasqualetti et al. 2016; Suardi et al. 2015; Ost et al. 2018). This might be supported by the results of our study, where men with only two positive iliacal sites had a lower risk of recurrence than men with more positive sites (20% vs 71%). It is possible that these sites were adequately treated by lymphadenectomy or by an extended field of radiation therapy, although a possible interpretation might also be that choline PET/CT scans with only one or two positive sites are at a higher risk of being false positive. Further randomized trials will be needed to evaluate the true therapeutic value of aggressively treating oligometastatic disease, especially for sites outside the template of an extended lymph node dissection.

Our study is, as far as we know, the first to evaluate the prognostic value of choline PET/CT for primary metastasis staging. Its strengths include the large number of consecutive patients and the long follow-up period (median 75 months). The limitations are chiefly the retrospective design and the low number of events, especially regarding CSM. However, recent data suggests that metastasis-free survival, which was significantly longer in this study, is a good surrogate for cancer-specific survival (Xie et al. 2017).

## Conclusions

In men with a newly diagnosed high-risk prostate cancer and a negative or inconclusive bone scan, ^18^F-choline uptake on PET/CT suggestive of metastasis was associated with recurrence, progression to distant metastasis, and prostate cancer death. This strongly indicates that the choline uptakes represented metastasis and not false positive findings.
